# Comparison study of anterior cervical zero-profile fusion cage (ROI-C) and traditional titanium plate plus fusion technique for the treatment of spinal cord type cervical spondylosis

**DOI:** 10.1097/MD.0000000000036651

**Published:** 2023-12-15

**Authors:** Haoran Gao, Zhen Tian, Yong Wang, Zhaohui Lou

**Affiliations:** a Department of the Orthopedic Surgery, the First Affiliated Hospital of Zhengzhou University, Zhengzhou University, Henan, China.

**Keywords:** anterior cervical zero-profile fusion cage (ROI-C), cervical spondylotic myelopathy, clinical comparative study, titanium plate with fusion cage

## Abstract

A retrospective comparative study. To compare and analyze the differences in the efficiency and safety of ROI-C and traditional titanium plate with fusion cage for the treatment of CSM patients. Clinical data of 105 patients with CSM who underwent surgical treatment at our hospital from January 2019 to December 2020 were retrospectively reviewed. Patients were divided into ROI-C and traditional groups according to the different fusion methods. The operation time, intraoperative blood loss, preoperative and postoperative JOA score, NDI score, cervical Cobb angle, intervertebral space height, and postoperative complications were recorded and compared between the 2 groups. A total of 105 patients were included in this study, with 57 patients in the ROI-C group and 48 patients in the traditional group. The baseline data were similar between the 2 groups (*P* > .05). The operative time, intraoperative blood loss, and the incidence of postoperative dysphagia were significantly lower in the ROI-C group than in the traditional group (*P* < .05). There were no significant differences in the JOA score, NDI score, cervical Cobb angle, intervertebral space height, the incidence of postoperative axial symptoms, and adjacent segment degeneration between the 2 groups (*P* > .05). However, both groups showed significant improvement in the JOA score, NDI score, cervical Cobb angle, and intervertebral space height compared with before surgery (*P* < .05). The ROI-C zero-profile internal fixation system and traditional titanium plates with fusion cages can achieve satisfactory clinical treatment results for CSM patients. However, ROI-C has advantages of a shorter operative time, less blood loss, and less postoperative dysphagia. Therefore, the ROI-C zero-profile internal fixation system can be safely and effectively used to treat patients with CSM.

## 1. Introduction

Cervical spondylotic myelopathy (CSM) is a prevalent cause of spinal cord injury in adults, affecting approximately 10% of patients aged 55 years and older.^[1]^ The condition can result from a variety of factors, including vertebral protrusion, bony redundancy, and ligament calcification, all of which can lead to progressive spinal stenosis and subsequent compression of the spinal cord and nerves.^[2]^ Symptoms tend to worsen over time, but the rate of deterioration is unpredictable and variable.^[3,4]^ Surgical intervention is the most common treatment for patients with cervical spondylosis and neurological symptoms.^[5,6]^ Studies have indicated that 20% to 60% of patients who receive conservative treatment experience disease progression within 3 to 6 years and may ultimately require surgery.^[7]^

Anterior cervical discectomy and fusion (ACDF) was first introduced by Smith and Cloward, who proposed anterior decompression and disc removal, typically involving the placement of endophytes in the intervertebral space and a titanium plate for fixation.^[8,9]^ ACDF has become the preferred surgical approach, given its favorable clinical outcomes, including effective decompression, restoration of intervertebral space height, improved neurological symptoms, and reconstruction of the cervical spine physiological curvature.^[10]^ However, ACDF can also result in complications such as dysphagia, pseudoarthrosis, and plate loosening and displacement.^[11]^ A U.S. Orthopaedic Surgery Database study found that up to 90% of surgeons use the anterior cervical approach for cervical spine treatment.^[12]^ Several studies have shown that ACDF combined with titanium plate fixation is safe and effective for treating multilevel cervical spondylotic myelopathy.^[13]^

In recent years, the anterior cervical zero-tangential fusion device (ROI-C) has emerged as a promising alternative in the field, and its use has become widespread.^[14]^ The ROI-C is a novel zero-tangential fusion device with 2 curved inserts that provide immediate stability and secure fixation while fully incorporating into the vertebral space. This design allows for initial firm fixation, which ensures excellent long-term treatment outcomes by enabling long-term fusion fixation. Although many clinical reports have described the use of ROI-C for the treatment of single-segment spinal cord spondylosis, there is a dearth of research on the effectiveness of the ROI-C in dual- and multisegmental cervical spondylosis. Therefore, we conducted a retrospective analysis of CSM patients who were treated in our hospital and underwent ROI-C internal fixation or fusion implantation with titanium plates to explore the effectiveness and safety of the ROI-C system for the treatment of CSM. The aim of this study was to compare and analyze the perioperative outcomes and complications of these 2 surgical techniques.

## 2. Materials and methods

This retrospective study was formally approved by the Ethics Committee of the First Affiliated Hospital of Zhengzhou University. Informed consent was waived because of the retrospective nature of the study and the use of de-identified patient data.

### 2.1. Sample inclusion

**Figure 1.**
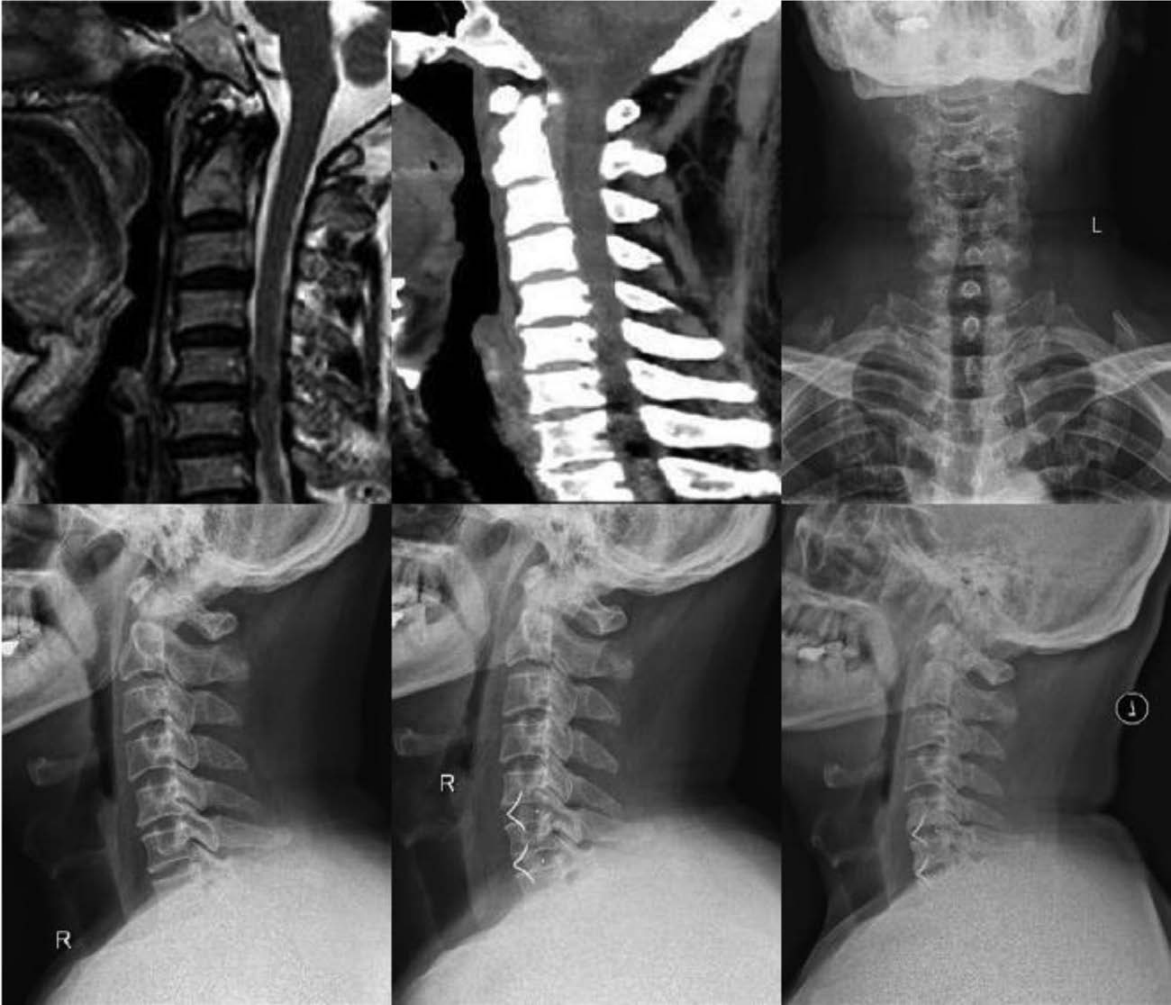
Anterior cervical discectomy ROI-C internal fixation in a patient with 2-segment spinal cord cervical spondylosis.

**Figure 2.**
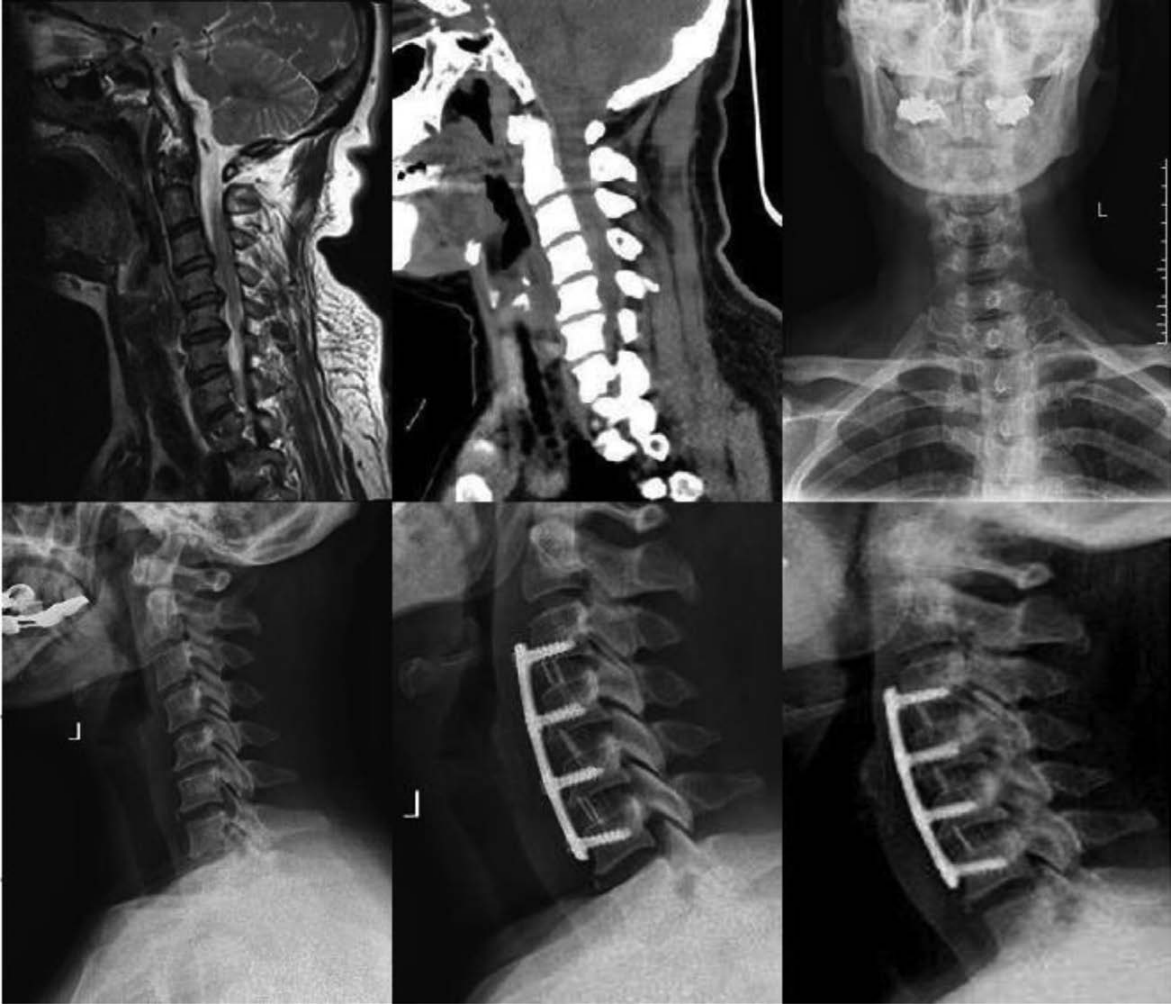
Anterior cervical discectomy fusion with titanium plate fixation in a patient with 3-segment spinal cord spondylolisthesis.

**Figure 3.**
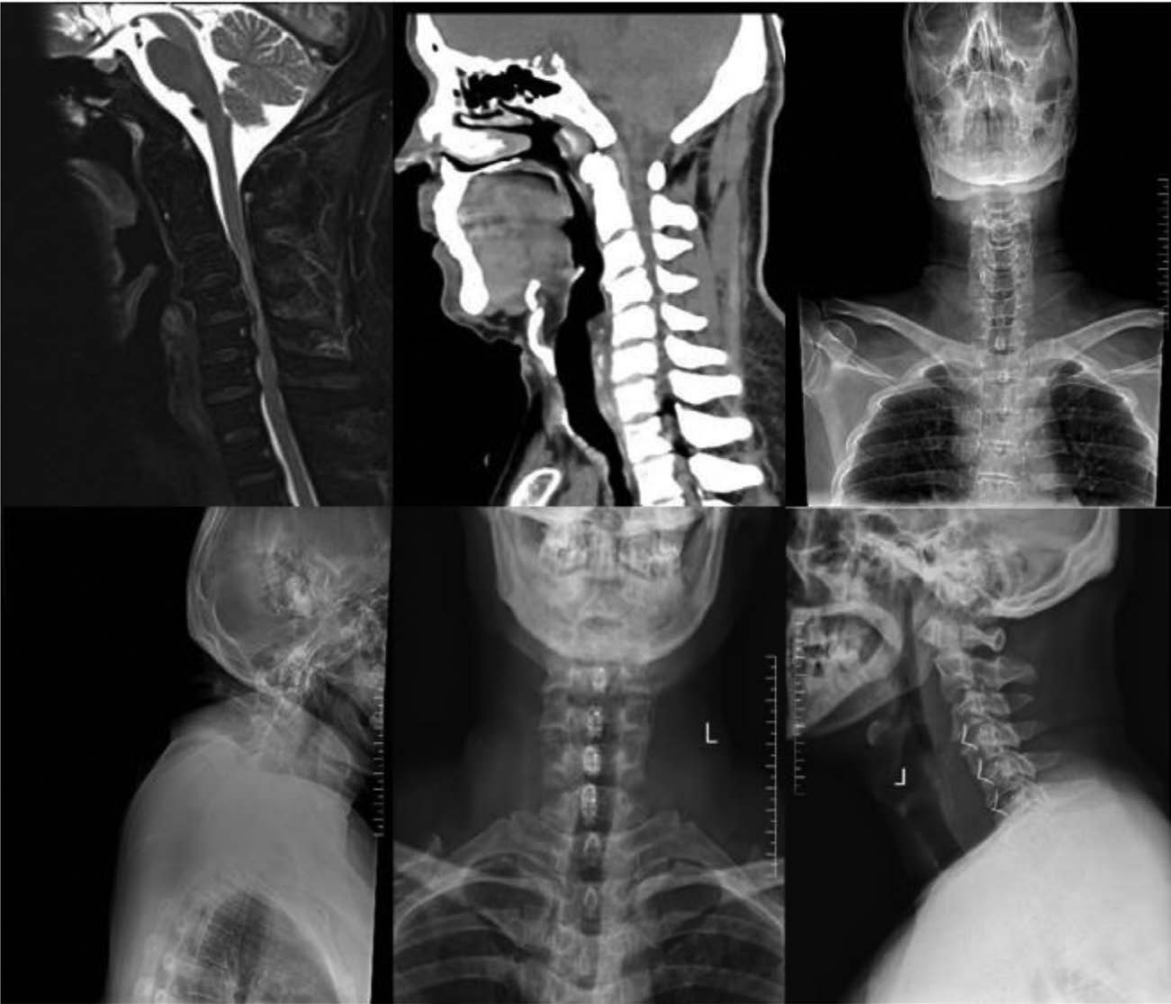
Anterior cervical discectomy ROI-C internal fixation in a patient with 4-segment spinal cord cervical spondylosis.

We reviewed the clinical data of 105 patients with cervical spondylosis who visited our hospital from January 2019 to December 2020. The patients were divided into 2 groups based on the surgical procedures performed: the ROI-C group, which included 57 patients who underwent anterior cervical discectomy and ROI-C internal fixation (26 males and 31 females; mean age, 58.40 ± 6.72 years; operated segments: single-segment, 24 cases; double-segment (Fig. 1), 22 cases; triple-segment (Fig. 2), 10 cases; and quadruple-segment (Fig. 3), 1 case), and the conventional group, which included 48 patients who underwent anterior cervical discectomy and fusion with titanium plate implantation (23 males and 25 females; mean age, 58.63 ± 7.39 years; operated segments: single-segment, 23 cases; double-segment, 18 cases; and triple-segment, 6 cases).

All patients exhibited signs and symptoms typical of cervical spondylotic myelopathy (CSM) on admission, including decreased manual dexterity, ascending sensory abnormalities in the upper or lower extremities, hyperreflexia in the heel and knee tendons, and positive Babinski and Hoffmann signs. The baseline data between the 2 groups were comparable and not significantly different (*P* > .05) (see Table 1).

**Table 1:** Comparison of baseline information between the 2 groups of patients.

Indicators	ROI-C group	Traditional group	t/χ²	*P*
Number of cases(n)	57	48	-	-
Gender [*n* (%)}				
Male	26 (45.6%)	23 (47.9%)	0.056	.814
Female	31 (54.4%)	25 (52.1%)		
Age (*x̅ ± s*, years)	58.40 ± 6.72	58.63 ± 7.39	0.161	.873
Smoking [*n* (%)}	22 (38.6%)	20 (41.7%)	0.102	.749
BMI (*x̅ ± s*, kg/m^2^)	23.33 ± 2.43	22.66 ± 2.19	1.477	.143
Surgical segment [*n* (%)}				
Single-segment	24 (42.1%)	23 (47.9%)	0.909	.872*
Two-segment	22 (38.6%)	18 (37.5%)		
Three-segment	10 (17.5%)	6 (12.5%)		
Four-segment	1 (1.8%)	1 (2.1%)		

* Fisher precision test was used

### 2.2. Data collection

All patients included in the study underwent cervical spine frontal, lateral, and powered DR films, cervical spine CT and MRI examinations. For patients with sensory abnormalities such as numbness and pain in the hands, electromyography was performed to differentiate CSM from other diseases. Frontal and lateral cervical spine radiographs showed varying degrees of cervical spine degeneration with normal or straightened physiological curvature, partial intervertebral joint space narrowing, or ossification of the posterior longitudinal ligament. MRI was used to assess spinal cord compression and the degree of spinal stenosis.

### 2.3. Inclusion and exclusion criteria

#### 2.3.1. Inclusion criteria.

Patients were included if they presented with the following:

Obvious symptoms of cervical spondylotic myelopathy (CSM), such as upper limb numbness, unsteadiness while walking, limb weakness, and a feeling of stepping on cotton.

Obvious signs of CSM, such as increased muscle tone, decreased muscle strength, hyperreflexia of the Achilles and knee tendons, or positive Babinski or Hoffmann sign.

Preoperative and postoperative radiographs, computed tomography (CT), and magnetic resonance imaging (MRI) examinations were complete and without image loss. Preoperative MRI of the neck indicated single or continuous double or multiple segment cervical medullary compression, and the imaging manifestations were consistent with the clinical symptoms and physical examination findings.

No serious cardiopulmonary disorders and the physical ability to tolerate the surgery.

### 2.4. Exclusion criteria

Patients were excluded if they met any of the following criteria:

History of cervical spine trauma or previous surgery, preoperative cervical spine tuberculosis, tumors, cervical instability, cervical deformity, and ossification of the posterior longitudinal ligament, or preoperative dysphagia.

Non-continuous multisegmental spinal cord compression.

Partial loss of examination images before and after surgery resulting in incomplete data or with postoperative follow-up shorter than 1 year.

Severe cardiopulmonary disorders that cannot tolerate anesthesia and surgery.

Psychological disorders that prevent the patient from cooperating with treatment and follow-up on their condition.

Amyotrophic lateral sclerosis, progressive polyarthritis, and spinal cord demyelination were also excluded.

### 2.5. Surgery method

**Figure 4.**
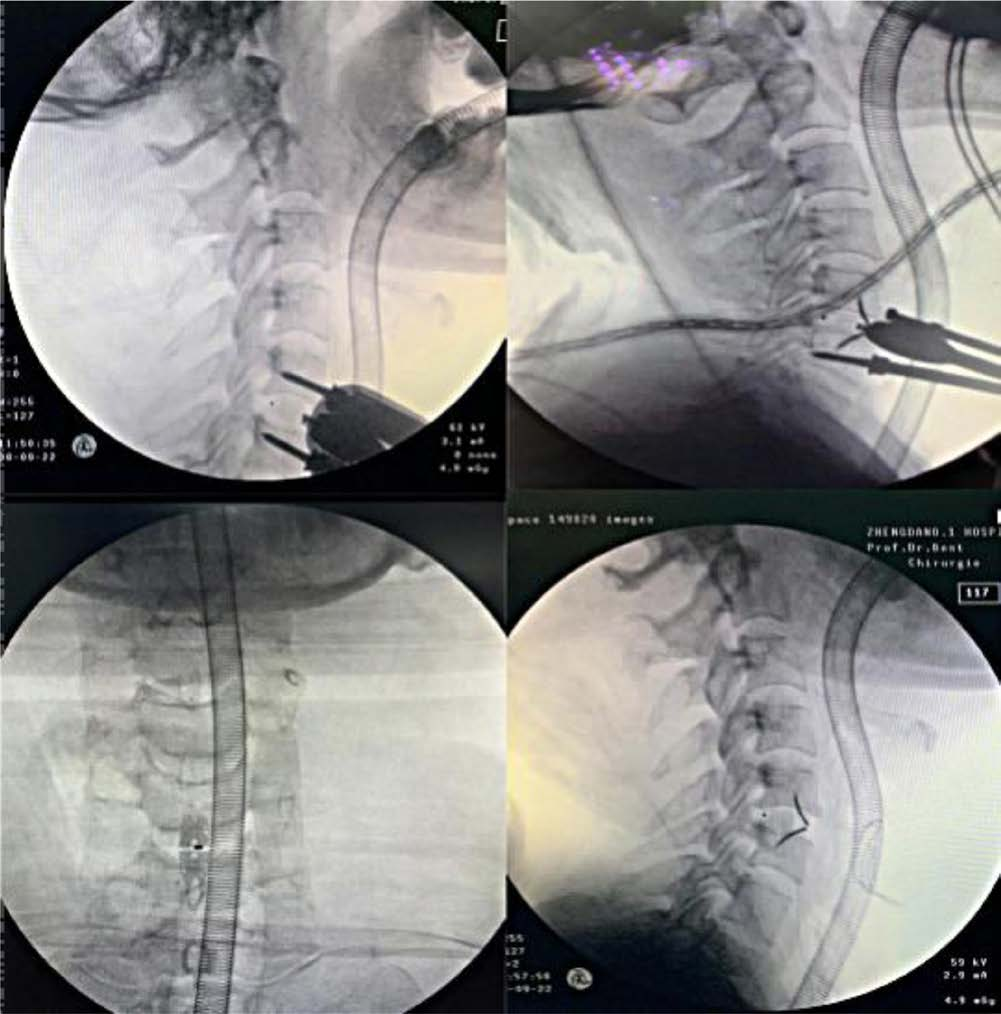
The figure shows the general flow of the single-stage operation under intraoperative C-arm fluoroscopy.

Both procedures were performed by the same team of surgeons. After anesthesia induction, the patient was positioned supine and routinely sterilized. C-arm fluoroscopy was utilized to locate and mark the target segment. A transverse incision was made on the right side of the anterior cervical area, followed by a blunt longitudinal dissection along the broad deep layer of the cervical muscle to expose the medial edge of the sternocleidomastoid muscle. Blunt separation was performed along the sternocleidomastoid muscle and tracheal space, with the carotid sheath and sternocleidomastoid muscle held laterally, and the tracheoesophageal sheath retracted medially to expose the prevertebral fascia and create a longitudinal incision. Intraoperative C-arm fluoroscopy was used again to clarify the responsible gap, and a spreader was utilized to open the vertebral space. A sharp knife was used to dissect the annulus fibrosus, and the nucleus pulposus was removed with forceps to relieve the cervical medullary compression, followed by removal of remnants with a spatula and posterior longitudinal ligament. Osteophytes at the vertebral edges and hook vertebral joints were removed using either a 1 mm vertebral plate biting forceps or a high-speed grinding drill. The upper and lower cartilage endplates were scraped with a spatula, up to the subchondral bone. The removed osteophytes were used as bone graft material, mixed with allograft bone, and co-filled in the fusion device. After confirming spinal cord decompression, the following steps were performed:

In the ROI-C group, the fusion device loaded with bone particles was placed in the appropriate position, and 2 curved clamps were alternately inserted into the fusion device after the spacer was withdrawn (Fig. 4).

In the Traditional group, the fusion device loaded with bone particles was placed in the appropriate position, the titanium plate was placed in front of the vertebral body and locked with screws after the spacer was withdrawn. Drainage was placed, and the incision was closed after C-arm fluoroscopy was used again to confirm the ideal position of internal fixation.

Preoperative tracheal nudge training was routinely performed for all patients, and postoperative rehabilitation measures were advised to enhance tolerance during the operation. Following surgery, patients were immediately placed in a cervical orthosis and transferred to the ward for cardiac monitoring, primary care, and oxygen inhalation as needed. Functional cervical and neurological rehabilitation was initiated immediately after surgery. Cervical frontal and lateral digital radiographs were obtained on the third postoperative day. Upon discharge, patients were instructed to wear a rigid cervical orthosis for 1 month followed by an inflatable cervical orthosis for 2 months. They were advised to gradually resume normal activities and to discontinue wearing the orthosis after 3 months. Patients included in the study underwent imaging review at 3 months and 1 year after surgery, and follow-up data were recorded using a standardized questionnaire.

## 3. Observed parameters

### 3.1. Perioperative data

Baseline information, operative time, and intraoperative bleeding were recorded and compared between the 2 study groups.

### 3.2. Clinical outcomes

The degree of neurological recovery was evaluated using the Japanese Orthopaedic Association (JOA) scores,^[15]^ which included assessments of sensory-motor function of the extremities, trunk, and bladder function. The final score ranged between 0 and 17, with lower scores representing poorer function. The improvement rate of the JOA score was calculated as follows: [(final follow-up JOA score - preoperative JOA score)/(17 - preoperative JOA score)] × 100%.

The degree of cervical spine dysfunction was assessed using the Neck Disability Index (NDI).^[16]^ The NDI consists of ten items evaluating pain intensity, living situation, lifting, reading, etc. Scores range from 0 to 50, with lower scores indicating less cervical dysfunction.

### 3.3. Statistical processing

Data were analyzed using SPSS version 20.0 (IBM Corporation, Armonk, NY, USA). Continuous variables were expressed as mean ± standard deviation (`x ± s), and repeated-measures ANOVA was used to compare changes in the JOA score, NDI score, cervical Cobb angle, and intervertebral space height at each follow-up time point before and after surgery in both groups. The t-test was used to compare between-group differences. Categorical variables were presented as frequencies and percentages, and the chi-square test was used for between-group comparisons. For theoretical frequencies between 1 and 5, the continuous corrected chi-square test was used, and for theoretical frequencies <1, the Fisher exact test was used. A *P* value of <.05 was considered statistically significant. Furthermore, statistical results were further demonstrated through graphic methods using R studio, including line figures and violin diagrams.

## 4. Results

### 4.1. Baseline information

Table 1 shows that there were no statistically significant differences between the 2 groups in terms of sex, age, smoking status, BMI, or surgical segment information (*P* > .05).

### 4.2. Surgical data

**Figure 5.**
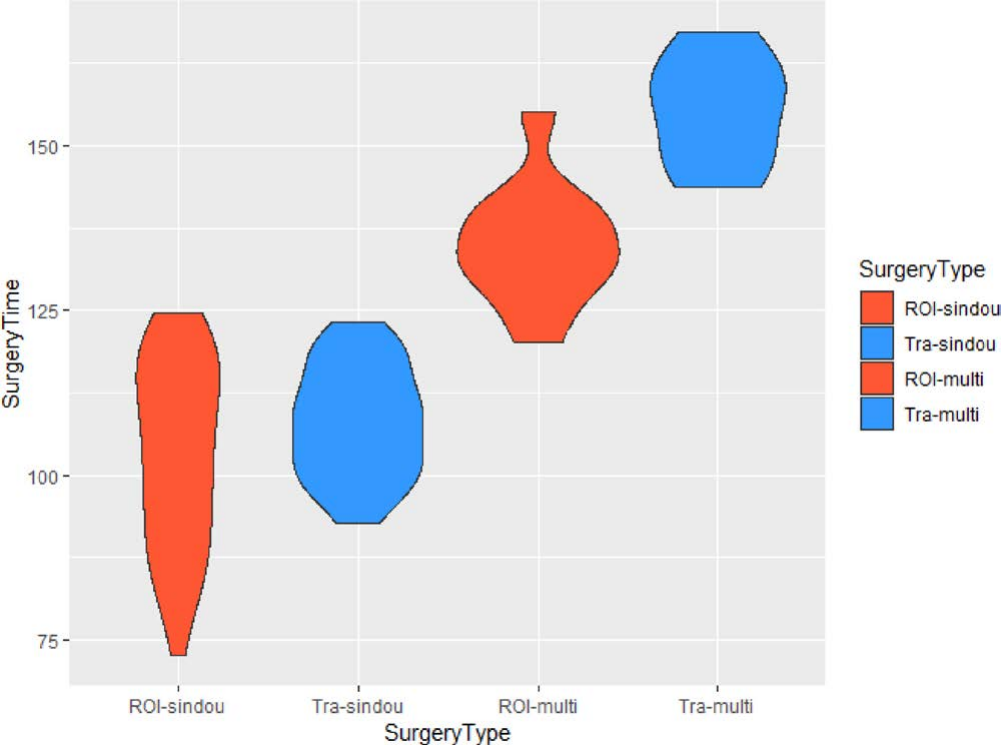
Comparison diagram of surgery time (min) between 2 groups of patients.

**Figure 6.**
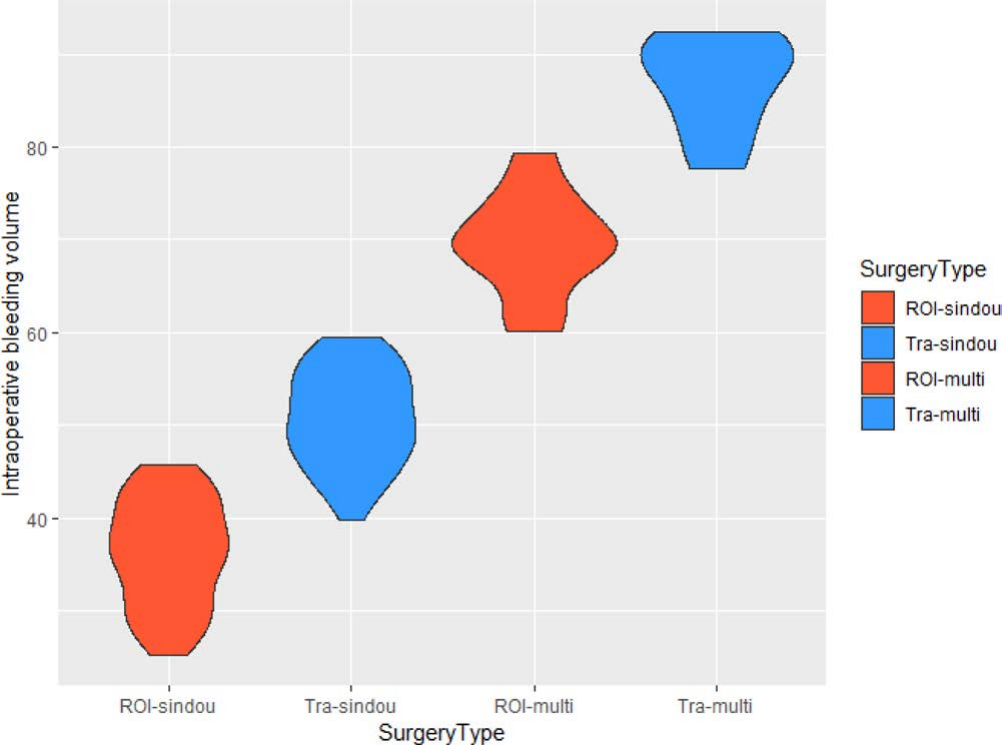
Comparison diagram of intraoperative bleeding volume (mL) between 2 groups of patients.

Operative time and intraoperative bleeding for single/dual or multiple segments were shorter in the ROI-C group compared to the conventional group, and the difference between the 2 groups was found to be significant (*P* < .05) (Refer to Table 2 for detailed information). Based on the graph in Figures 5 and 6, generated according to Table 2, it becomes more visually apparent to observe the aforementioned results.

**Table 2:** Comparison of surgical data between 2 groups of patients.

Group	Number of cases (n)	Operating time (*x̅ ± s*, min)		Intraoperative bleeding volume (*x̅ ± s*, mL)	
		Single/double section	Multi-segment	Single/double section	Multi-segment
ROI-C group	57	104.04 ± 19.55	133.27 ± 14.06	36.63 ± 10.22	65.55 ± 12.18
Traditional group	48	114.39 ± 20.37	154.71 ± 14.57	46.24 ± 11.22	84.71 ± 12.93
t	-	2.416	3.112	4.182	3.015
*P*	-	.018	.007	<.001	.008

### 4.3. Clinical efficacy

**Figure 7.**
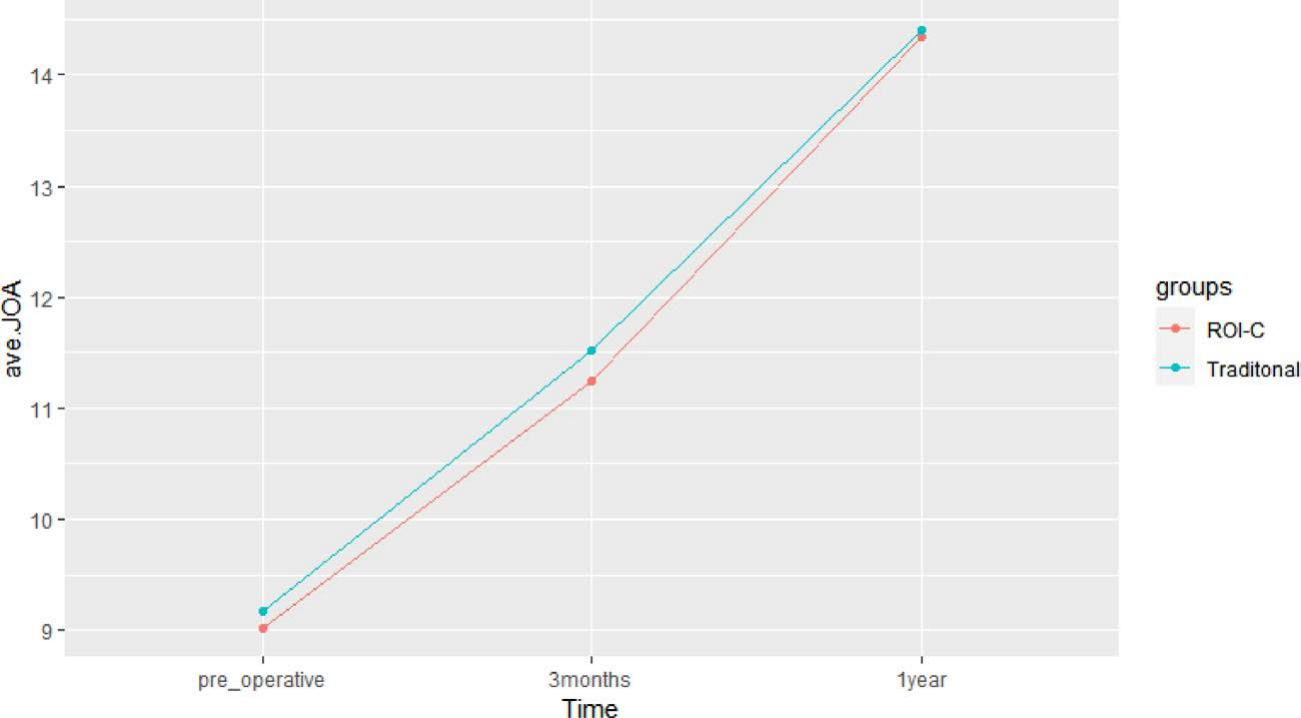
Changes in JOA scores in the 2 groups. JOA = Japanese orthopedic association scores.

**Figure 8.**
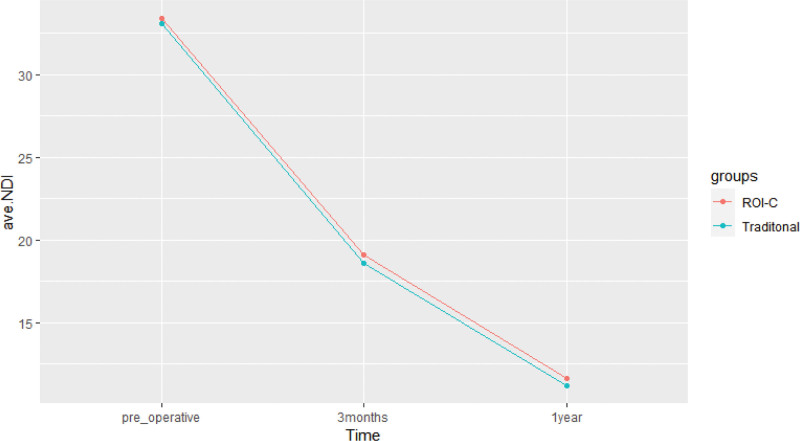
Changes in NDI scores in the 2 groups. NDI = neck disability index.

At 3 months and 1 year postoperatively, there was a significant improvement in JOA and NDI scores for patients in both the ROI-C and conventional groups compared to their preoperative scores (*P* < .001). However, there were no significant differences in JOA and NDI scores between the 2 groups before surgery and at the follow-up time points of 3 months and 1 year postoperatively (*P* > .05) (Refer to Table 3, Fig. 7, Table 4, and Fig. 8 for further details).

**Table 3:** Changes in JOA scores in the 2 groups (` x ± s, points).

Group	Number of cases (n)	Pre-operative	3 mo after surgery	1 yr after surgery	JOA improvement rate (%)
ROI-C group	57	9.02 ± 1.42	11.25 ± 1.54	14.35 ± 1.14	67.67 ± 11.56
Traditional group	48-	9.17 ± 1.53	11.52 ± 1.49	14.40 ± 1.07	67.14 ± 11.33
t	-	0.517	0.927	0.207	0.234
*P*	-	.607	.356	.836	.815

**Table 4:** Changes in NDI scores in the 2 groups (` x ± s, points).

Group	Number of cases (n)	Pre-operative	3 mo after surgery	1 yr after surgery
ROI-C group	57	33.39 ± 3.12	19.07 ± 1.78	11.58 ± 1.83
Traditional group	48	33.10 ± 2.97	18.60 ± 2.30	11.17 ± 1.48
t	-	0.471	1.168	1.253
*P*	-	.639	.245	.213

### 4.4. Imaging evaluation

**Figure 9.**
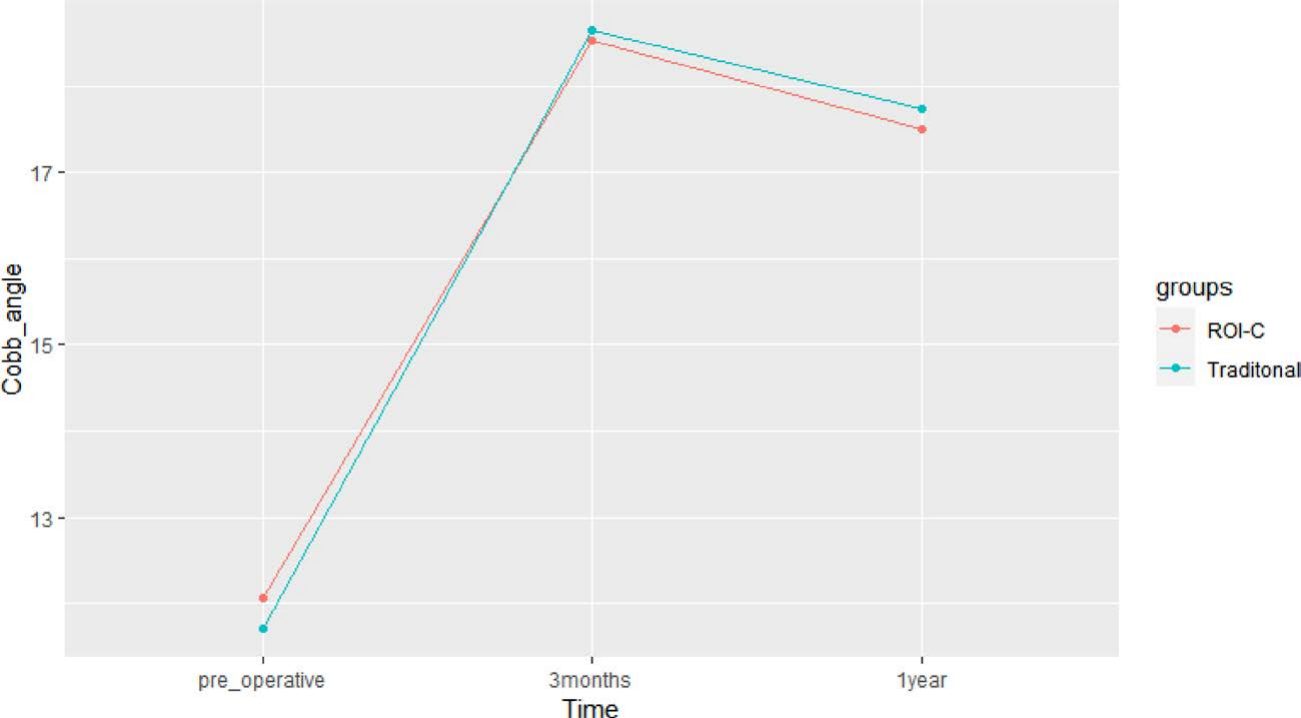
Changes in the Cobb angle of the cervical spine in the 2 groups.

**Figure 10.**
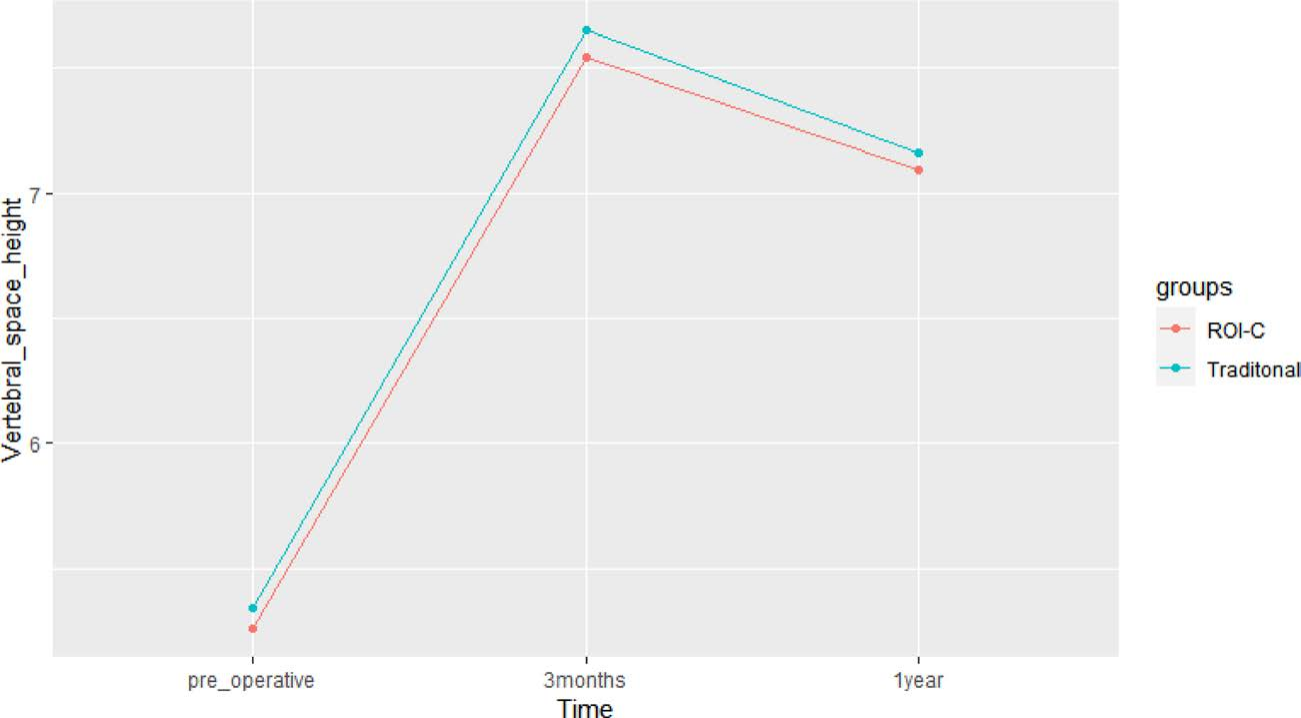
Changes in vertebral space height in the 2 groups.

1. The Cobb angle of the cervical spine was measured and analyzed in both groups. In the ROI-C group, the preoperative Cobb angle was (12.07 ± 3.47)°, which improved to (18.52 ± 3.18)° at 3 months and (17.51 ± 3.20)° at 1 year after surgery. There was a significant improvement in the Cobb angle at 3 months and 1 year after surgery compared to preoperatively (F = 578.640, *P* < .001). Similarly, in the conventional group, the preoperative Cobb angle was (11.71 ± 2.84)°, which improved to (18.64 ± 2.71)° at 3 months and (17.74 ± 2.67)° at 1 year after surgery. There was a significant improvement in the Cobb angle at 3 months and 1 year after surgery compared to preoperatively (F = 669.626, *P* < .001). However, there were no significant differences in the Cobb angle between the ROI-C group and conventional group at the preoperative, 3-month, and 1-year postoperative follow-up time points (*P* > .05) (See Table 5 and Fig. 9).
2. Intervertebral space height was evaluated in 2 groups of patients undergoing spinal surgery. In the ROI-C group, the mean intervertebral space height was (5.26 ± 0.73) mm preoperatively, (7.54 ± 0.82) mm at 3 months postoperatively, and (7.09 ± 0.84) mm at 1 year postoperatively. While a partial decrease in height was observed at 1 year postoperatively, intervertebral space height significantly improved at 3 months and 1 year postoperatively compared to preoperative levels (F = 1296.631, *P* < .001). Similarly, in the conventional group, the mean intervertebral space height was (5.34 ± 0.68) mm preoperatively, (7.65 ± 0.81) mm at 3 months postoperatively, and (7.16 ± 0.88) mm at 1 year postoperatively. While a partial decrease in height was observed at 1 year postoperatively, intervertebral space height significantly improved at 3 months and 1 year postoperatively compared to preoperative levels (F = 1044.009, *P* < .001). There was no significant difference in intervertebral space height between the 2 groups at the preoperative, 3-month, and 1-year postoperative follow-up points (*P* > .05) (See Table 6 and Fig. 10).
3. Regarding intervertebral fusion, a total of 55 patients in the ROI-C group achieved bony fusion at 3 months postoperatively, with a fusion rate of 96.5%, and 57 patients achieved bony fusion at 1 year postoperatively, with a fusion rate of 100%. Similarly, in the conventional group, 45 patients achieved bony fusion at 3 months postoperatively, with a fusion rate of 93.8%, and 48 patients achieved bony fusion at 1 year postoperatively, with a fusion rate of 100%. No significant differences in intervertebral fusion rates were observed between the 2 groups at the 3-month and 1-year postoperative follow-up time points (*P* > .05).

**Table 5:** Changes in the Cobb angle of the cervical spine in the 2 groups (` x ± s, °).

Group	Number of cases (n)	Pre-operative	3 mo after surgery	1 yr after surgery
ROI-C group	57	12.07 ± 3.47	18.52 ± 3.18	17.51 ± 3.20
Traditional group	48	11.71 ± 2.84	18.64 ± 2.71	17.74 ± 2.67
t	-	0.581	0.200	0.390
*P*	-	.562	.842	.697

**Table 6:** Changes in vertebral space height in the 2 groups (` x ± s, mm).

Group	Number of cases (n)	Pre-operative	3 mo after surgery	1 yr after surgery
ROI-C group	57	5.26 ± 0.73	7.54 ± 0.82	7.09 ± 0.84
Traditional group	48	5.34 ± 0.68	7.65 ± 0.81	7.16 ± 0.88
t	-	0.599	0.710	0.390
*P*	-	.550	.479	.698

### 4.5. Complications

Dysphagia: Four patients in the ROI-C group experienced mild dysphagia after surgery, with an incidence rate of 7.0% (4/57), and their symptoms disappeared after 3 months of conservative treatment. In contrast, dysphagia occurred in 14 patients in the conventional group, with 9 cases being mild, 4 cases being moderate, and 1 case being severe. The incidence rate of dysphagia was 29.2% (14/48), and 3 patients still had dysphagia at the 3-month follow-up, including 2 mild cases and 1 moderate case. However, all patients’ dysphagia symptoms were relieved at the 1-year follow-up. The overall incidence of dysphagia was significantly lower in the ROI-C group than in the conventional group (*χ*²=9.000, *P* = .003).

Axial symptoms: Three patients in the ROI-C group experienced axial symptoms after surgery, which were characterized by neck and shoulder pain and discomfort, and were adequately managed with painkillers. The incidence rate of axial symptoms was 5.3% (3/57), and all symptoms disappeared after 3 months of conservative treatment. In the conventional group, 4 patients had axial symptoms after surgery, with 1 case showing neck pain and the remaining 3 cases showing neck and shoulder pain and discomfort, which were also managed with painkillers. The incidence rate of axial symptoms was 8.3% (4/48), and 1 patient still had axial symptoms after 3 months of conservative treatment, characterized by neck and shoulder pain with stiffness. However, all patients’ axial symptoms disappeared at the 1-year follow-up. There was no significant difference in the overall incidence of axial symptoms between the 2 groups (χ²=0.056, ^#^ *P* = .814) (^#^ using the continuous corrected chi-square test).

Degeneration of adjacent segments: At 3 months and 1 year postoperatively, no adjacent segment degeneration (ASD) was detected in patients in the ROI-C group. In the conventional group, no ASD was observed at 3 months postoperatively; however, 2 patients (4.2%, 2/48) had ASD at 1 year postoperatively. The difference in the incidence of ASD between the 2 groups was not statistically significant and was comparable. (*P = .207) (*Fisher exact test was used).

## 5. Discussion

In this study, we recorded and compared operative time and intraoperative bleeding between 2 groups, the ROI-C group and the conventional group. Our findings showed that the ROI-C group had significantly shorter operative time and less intraoperative bleeding compared to the conventional group. We attribute these results to the relative simplicity of the implantation process and fixation method of the ROI-C system compared to traditional fusion implantation with titanium plate fixation. Specifically, the small opening in front of the ROI-C fusion allowed for a curved insert to pass through and into the guiding channel, which could then be firmly locked with a percussion device. This allowed the ROI-C group to complete the fusion implantation and fixation operation by only exposing the lesion gap, whereas the conventional group required exposing the upper and lower vertebrae of the lesion gap, grinding and removing anterior vertebral redundancy, and performing screw drilling and tapping prior to titanium plate implantation.

In conclusion, our study demonstrates that the ROI-C system is associated with a more minimally invasive surgery, resulting in shorter operative time, less intraoperative bleeding, and lower risk of esophageal injury. These findings are consistent with previous studies, such as Zhou et al^[17]^ After analyzing the preoperative and postoperative JOA scores with NDI scores, it was observed that patients who underwent ROI-C internal fixation or fusion titanium plate fixation exhibited significant improvements in trunk and limb sensorimotor function and bladder function after surgery. Additionally, the degree of cervical spine impairment was reduced. The surgical treatment principles were the same in both groups, indicating that both procedures achieved satisfactory and similar clinical outcomes for CSM. However, no statistically significant difference between the groups was observed at each follow-up time point (*P* > .05). These findings are consistent with the results reported by He et al^[18]^ Nassr et al^[19]^ reported that mislocalized disc placement, in which the positioning needle is inserted into the standard disc, during anterior cervical surgery may increase the risk of adjacent segment degeneration (ASD). This finding suggests that intraoperative damage to the nucleus pulposus should be avoided. In our study, the incidence of intraoperative mislocalization was very low in both groups, and only a few cases of adjacent segment degeneration occurred in the conventional group. Thus, our results suggest that long-term irritation of the titanium plate is more likely the cause of ASD. Additionally, 2 cases in the traditional group experienced ASD at 1 year after surgery, with mild shoulder and neck pain. In these cases, patients were instructed to wear an inflatable neck brace for a specified duration, and the results indicated a significant improvement in both pain and neck function scores. The use of the inflatable neck brace has been demonstrated to provide support and stability to the cervical spine, thereby reducing cervical spine movement and pain associated with various conditions, such as cervical spondylosis, whiplash, and neck muscle strain. This finding supports the potential use of inflatable neck braces as a conservative management option for individuals with cervical spine-related disorders.

Gore^[20]^ reported that the cervical lordosis angle in normal people ranges from approximately 16° to 27°, which tends to increase with age and varies by sex. In this study, the preoperative cervical Cobb angle was (12.07 ± 3.47)° in the ROI-C group and (11.71 ± 2.84)° in the conventional group. The preoperative physiological curvature of the cervical spine was straightened to varying degrees in both groups, possibly due to intervertebral space narrowing caused by disc degeneration and the resultant decrease in the Cobb angle of the cervical spine. Reconstruction of the cervical spine structure led to increased cervical Cobb angle and interbody height in both groups at 3 months and 1 year after surgery. In the ROI-C group, fusion had an anterior-high and posterior-low morphology, which better maintained cervical convexity and interbody height after placement in the interspace. The conventional group used a pre-curved titanium plate to fix and lift the vertebral body in addition to placing the fusion in the interspace to maintain height, effectively restoring cervical curvature.^[21]^ However, both groups exhibited a partial decrease in cervical curvature at 1 year after surgery compared to 3 months after surgery, likely due to loss of interbody height. Fusion subsidence occurred when the loss of intervertebral space height in the operated segment reached 3 mm.^[22]^ At 1-year postoperative follow-up, both groups showed slight loss of intervertebral height in the surgical segment, but did not meet the criteria for fusion subsidence. A related study^[23]^ suggested that slight fusion subsidence after anterior cervical surgery can help maintain internal stability, but excessive subsidence should be avoided as it often leads to segmental instability. Cerier et al^[24]^ reported that smoking could have a negative impact on the rate of intervertebral fusion. In this study, all patients achieved intervertebral bone healing at the 1-year postoperative follow-up, which is consistent with the high fusion rates reported by Grasso G et al^[25]^ for both surgical approaches. The conventional group achieved a high fusion rate due to the fixation of the titanium plate, while the unique structural design of the ROI-C group contributed to its high fusion rate. The ROI-C system comprises a polyether ether ketone (PEEK) fusion device and 2 curved fixation inserts. PEEK is a non-absorbable biopolymer that offers biocompatibility, radiolucency, and an elastic modulus similar to that of human bone.^[26]^ Radiographic penetration enables the assessment of bone growth through the fusion device, while the material elastic modulus helps reduce stress shielding and promotes intervertebral fusion. The ROI-C fusion device also features a window that allows for autogenous bone filling or allogeneic bone grafting to accelerate bone healing after implantation. The fixation insert has a “barb”-like self-locking anti-exit device that limits the horizontal deflection of the fusion and enables complete integration into the vertebral space for fixation without the need for additional locking with a titanium plate, thereby reducing the probability of tissue irritation. Moreover, the serrated structure on the fusion surface provides better initial stability.

The current study found that all patients achieved intervertebral bone healing at the 1-year postoperative follow-up, regardless of smoking status. However, it is important to note that all patients who had not achieved intervertebral fusion at 3 months postoperatively were smokers, and they all attained bony fusion at the 1-year postoperative follow-up after quitting smoking. Thus, this study supports the notion that smoking may lead to a decrease in intervertebral fusion rates, and it emphasizes the importance of smoking cessation to promote successful surgical outcomes.

## 6. Conclusions

The results of this study indicate that the anterior zero-incision cervical fusion (ROI-C) and traditional titanium plate and fusion technique can both achieve satisfactory clinical outcomes for patients with CSM. However, the ROI-C zero-incision internal fixation system has advantages over the traditional technique, including shorter operative time, less bleeding, and less postoperative dysphagia. Thus, the ROI-C system can be safely and effectively used to treat dual- and multisegmental cervical spondylosis.

### 6.1. Limitations

Several limitations of this study should be considered when interpreting the results. First, individual pain thresholds and requirements for the extremities and trunk vary, and surgical efficacy may not be accurately reflected due to differences in individual subjective sensations. Second, the small sample size and short follow-up period limit the accuracy of the long-term outcomes of the 2 surgical options for patients with cervical spinal spondylosis. Finally, the retrospective design of this study does not provide the same level of evidence as a prospective randomized trial with a larger sample size, and further research is needed to confirm the results of this study.

## Author contributions

**Conceptualization:** Haoran Gao, Zhaohui Lou.

**Data curation:** Haoran Gao.

**Methodology:** Zhen Tian.

**Project administration:** Yong Wang.

**Writing – original draft:** Haoran Gao.

**Writing – review & editing:** Zhaohui Lou.
